# Growth Suppression of Mouse Pituitary Corticotroph Tumor AtT20 Cells by Curcumin: A Model for Treating Cushing's Disease

**DOI:** 10.1371/journal.pone.0009893

**Published:** 2010-04-13

**Authors:** Madhavi Latha Yadav Bangaru, Jeffrey Woodliff, Hershel Raff, Sanjay Kansra

**Affiliations:** 1 Department of Endocrinology, Metabolism & Clinical Nutrition, Aurora St. Luke's Medical Center, Milwaukee, Wisconsin, United States of America; 2 Department of Pediatrics, Aurora St. Luke's Medical Center, Milwaukee, Wisconsin, United States of America; 3 Endocrine Research Laboratory, Aurora St. Luke's Medical Center, Milwaukee, Wisconsin, United States of America; 4 Department of Pharmacology, Medical College of Wisconsin, Milwaukee, Wisconsin, United States of America; Sun Yat-Sen University, China

## Abstract

**Background:**

Pituitary corticotroph tumors secrete excess adrenocorticotrophic hormone (ACTH) resulting in Cushing's disease (CD). Standard treatment includes surgery and, if not successful, radiotherapy, both of which have undesirable side effects and frequent recurrence of the tumor. Pharmacotherapy using PPARγ agonists, dopamine receptor agonists, retinoic acid or somatostatin analogs is still experimental. Curcumin, a commonly used food additive in South Asian cooking, has potent growth inhibitory effects on cell proliferation. Our laboratory recently demonstrated that curcumin inhibited growth and induced apoptosis in prolactin- and growth hormone-producing tumor cells [1]. Subsequently, *Schaaf et.al.* confirmed our findings and also showed the *in vivo* effectiveness of curcumin to suppress pituitary tumorigenesis. However the molecular mechanism that mediate this effect of curcumin are still unknown.

**Principal Findings:**

Using the mouse corticotroph tumor cells, AtT20 cells, we report that curcumin had a robust, irreversible inhibitory effect on cell proliferation and clonogenic property. The curcumin-induced growth inhibition was accompanied by decreased NFκB activity. Further, curcumin down-regulated the pro-survival protein Bcl-xL, depolarized the mitochondrial membrane, increased PARP cleavage, which led to apoptotic cell death. Finally, curcumin had a concentration-dependent suppressive effect on ACTH secretion from AtT20 cells.

**Conclusion:**

The ability of curcumin to inhibit NFκB and induce apoptosis in pituitary corticotroph tumor cells leads us to propose developing it as a novel therapeutic agent for the treatment of CD.

## Introduction

Pituitary tumors, although not generally metastatic in nature, do result in morbidity due to both altered hormonal patterns as well as side effects of therapy [2]. Pituitary corticotroph tumors secrete excess ACTH resulting in CD. The progression of CD is accompanied by several pathological conditions including diabetes, osteoporosis and hypertension [3]. To date no standard reliable medical therapy exists to decrease ACTH secretion in CD. The commonly accepted approach for treatment is **s**till pituitary surgery followed by radiation, and disease relapse is a common outcome with both. Medical therapies are still experimental with approaches to suppressing ACTH secretion including, D2R agonists, somatostatin receptor antagonist, thiazolidinediones (PPARγ agonists) and retinoic acid [4]–[7].

Increased expression of the pro-survival protein, Bcl-2 is a common occurrence in pituitary tumors [8]. The pro-survival Bcl-2 family of proteins (Bcl-2, Bcl-xL and Mcl-1), are target genes of NFκB, and confer resistance to mitochondrial apoptosis. For example neuronal cells overexpressing Bcl-2 fail to undergo dopamine-induced apoptosis [9].

Curcumin, in addition to being a food additive, has been used as a medicinal agent in the ancient Indian system of *Ayurvedic* medicine. It is a biphenolic compound, derived from the plant *Curcuma longa*, of the *Zingiberaceae* (ginger) family, and imparts the distinct yellow color to Indian curries. In the Indian population, it is estimated that the average daily consumption of curcumin is 60–100 mg [10]. It is now well accepted that one of the mechanisms by which curcumin suppresses tumor growth is by inhibiting constitutively activated NFκB [11]. Currently, the anti-tumor properties of curcumin are being evaluated in several clinical trials, including pancreatic and colon cancer and also for Alzheimer disease [12]. The selectivity of curcumin to target tumor cells, as demonstrated by its ability to induce apoptosis in hepatocellular carcinoma while having no effect on normal hepatocytes, makes it an attractive pharmacotherapeutic agent [13], [14]. Further, in Phase I clinical trials in humans, curcumin was tolerated up to 8000 mg/day [15].

We recently demonstrated that curcumin was effective at suppressing the proliferation of prolactin- and growth hormone- producing pituitary tumor cells [1]. A recent report confirmed our original *in vitro* observations and further demonstrated the *in vivo* effectiveness of curcumin to suppress pituitary tumorigenesis in both a xenograft tumor model as well as in primary cell cultures of human pituitary tumors [16]. However, the molecular mechanism by which curcumin induces apoptosis in pituitary tumor cells remains unknown.

In the present study we examined the growth suppressive effect of curcumin on a mouse corticotroph tumor cell line, AtT20 cells. We report that curcumin inhibits constitutively active NFκB, decreases expression of pro-survival protein Bcl-xL, resulting in mitochondrial apoptosis. In addition curcumin potently suppressed ACTH secretion. The ability of curcumin to suppress proliferation as well as attenuate hormone secretion, leads us to propose developing curcumin as a novel therapeutic agent in the management of CD.

## Results

### Curcumin suppresses cell proliferation and clonogenic ability of AtT20 cells

**Figure 1:**
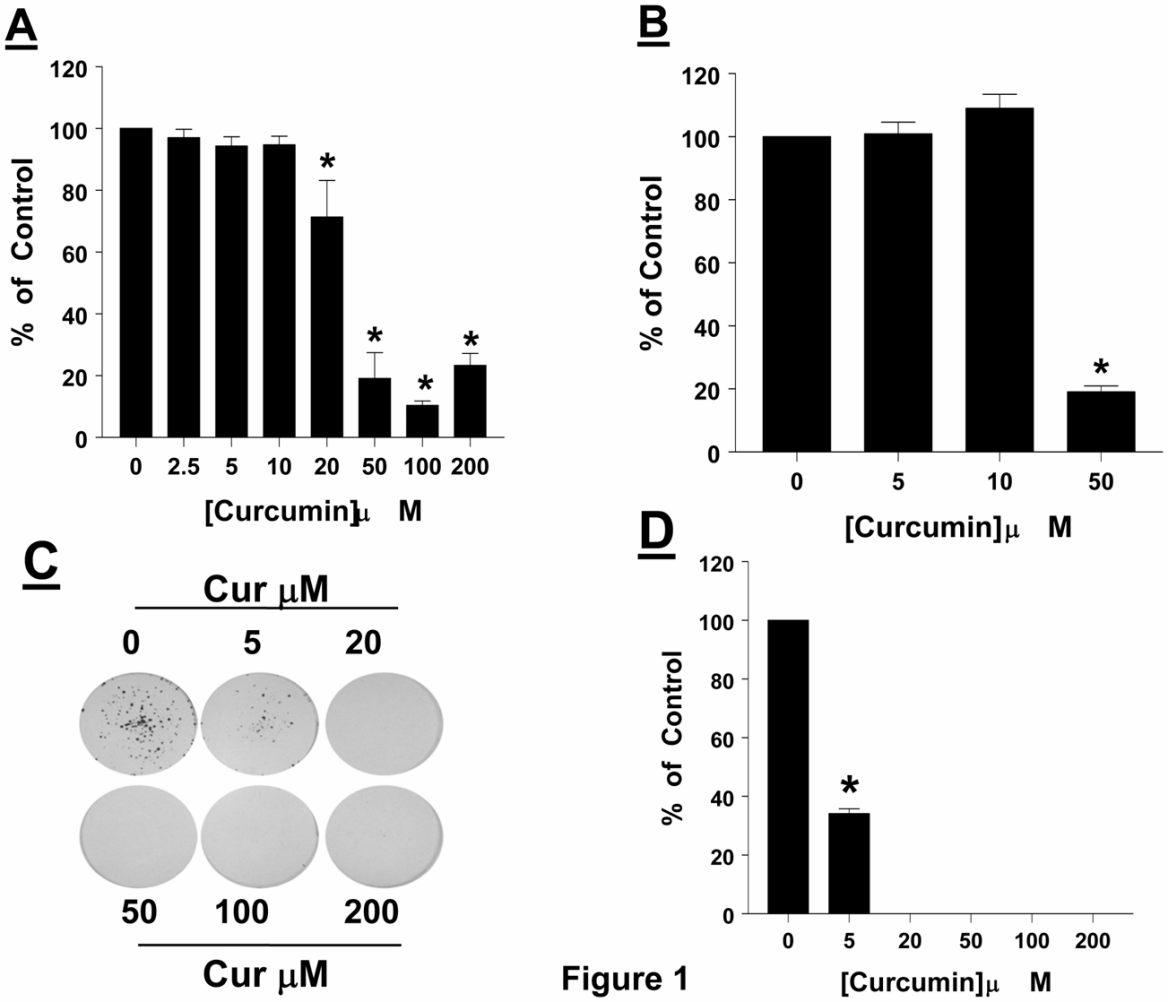
Curcumin suppresses cell proliferation and clonogenic ability of AtT20 cells. (*A*) AtT20 cells were treated with the indicated concentrations of curcumin and cell proliferation was determined by MTT assay after 4 days. Data were calculated as % of vehicle control and expressed as mean ±SEM of 3 independent experiments each performed with at least 4 replicates. * indicates significant differences from control, (*p<0.05*). (*B*) AtT20 cells were treated with either vehicle or curcumin (5, 10 and 50 µM) for 24 hrs. Cells were trypsinized, washed and re-plated in DMEM medium containing 10% FBS. Cell proliferation was assessed after 4 days. Data were calculated as % of vehicle control and expressed as mean ±SEM of 3 independent experiments each performed with at least 4 replicates. * indicates significant differences from control (*p<0.05*). (*C*) AtT20 cells were seeded (1000–3000 cells/well) in a six well plate in complete growth medium containing 10% FBS. Cells were allowed to adhere for 24 hrs, after which medium was replaced with fresh growth medium, containing 10% FBS, together with the indicated concentrations of curcumin. Medium was changed every 3 to 4 days, and colony formation was monitored over a 14–21 day period. Colony formation was detected by crystal violet staining and subsequently photographed. Data shown is from a single experiment, and is a representative of 3 independent experiments yielding similar results. (*D*) To quantitate colony formation, the number of colonies (each colony consisting of 50 cells or more) in 4 random fields were counted. The average was obtained, and decreases from 0 µM curcumin were calculated and are expressed as % of control. Each value is the mean ±SEM of 3 separate experiments. * indicates significant difference from control, (*p<0.05*).

We first examined the effect of curcumin on AtT20 cell proliferation. AtT20 cells were treated with curcumin (2.5–200 µM) and cell proliferation was assessed after 4 days. Our results show (Fig. 1A) that in a concentration-dependent manner curcumin suppressed AtT20 cell proliferation. Significant inhibition (28.68%; p<0.005) of cell proliferation was observed with 20 µM, and maximal inhibition (89.63%; p<0.005) being observed with 100 µM curcumin. We next questioned whether the growth inhibitory effect of curcumin on corticotroph tumor cell proliferation persisted upon removal of curcumin. AtT20 cells were treated with either vehicle or (5, 10 and 50 µM) curcumin for 24 hrs, after which cells were trypsanized, counted and equal number of cells from each treatment group were cultured in growth medium (containing 10% FBS) for an additional 4 days. Our results show (Fig. 1B) that an exposure to low concentrations of curcumin demonstrated proliferation that was similar to vehicle control. However, when AtT20 cells were exposed to 50 µM curcumin, they failed to proliferate in growth medium. These results suggest that curcumin induced growth suppression is irreversible.

We next questioned whether curcumin would have any effect on the clonogenic property of AtT20 cells. AtT20 cells were seeded (1000–3000 cells/well) in complete growth medium and allowed to adhere for 24 hrs. The medium was then replaced with complete growth medium containing the indicated concentrations of curcumin, and ability of AtT20 cells to form colonies was monitored over the next 14–21 days. Our results show (Fig. 1C) that the ability of AtT20 cells to form colonies is decreased in presence of curcumin. Significant inhibition (65.86%; p<0.05) of colony formation was detected with 5 µM curcumin from 20 µM curcumin concentrations, colony formation was completely abolished (Fig. 1D).

### Curcumin suppresses constitutively activated NFκB activity, down regulates Bcl-_x_l and causes mitochondrial membrane depolarization in AtT20 cells

**Figure 2:**
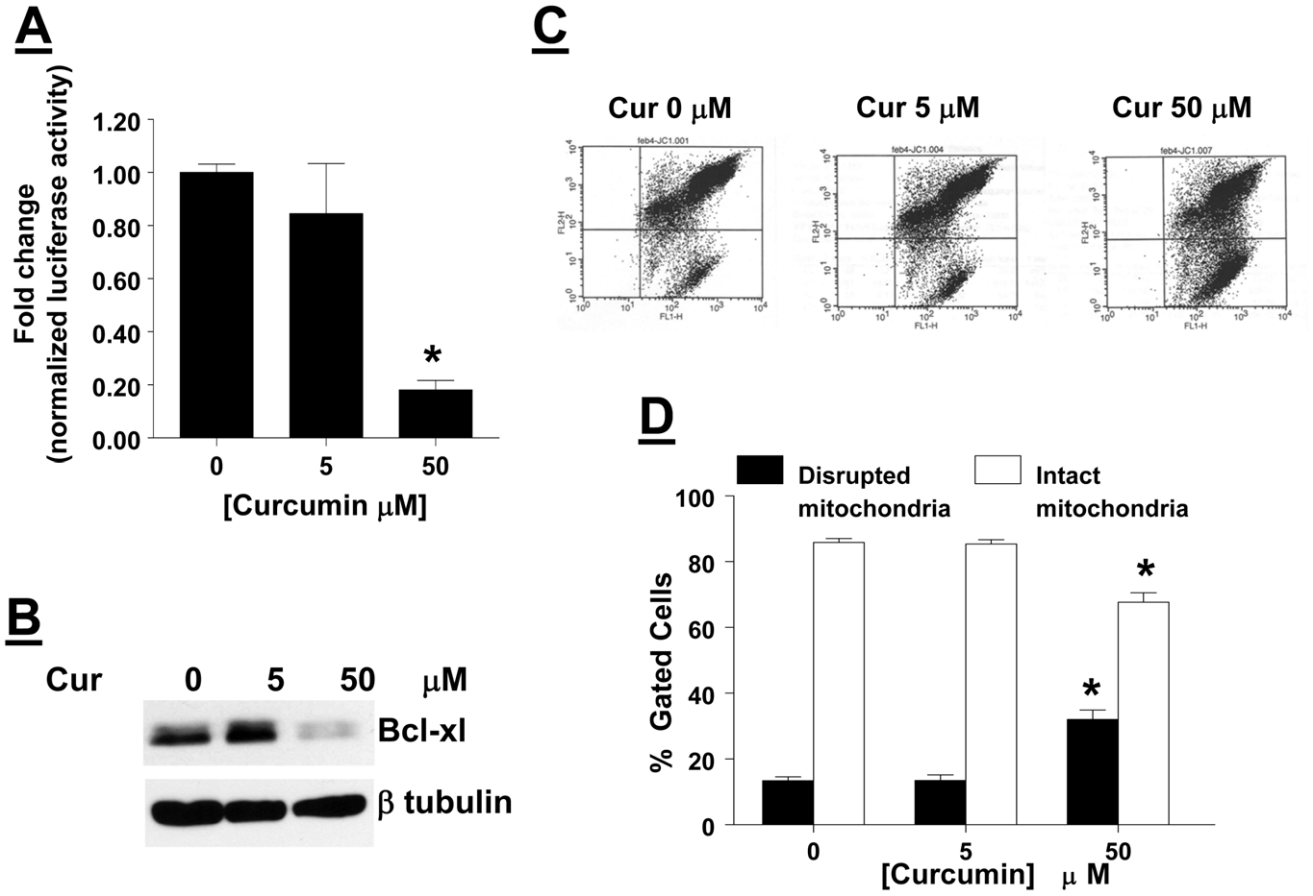
Curcumin suppresses constitutively activated NFκB activity, down regulates Bcl-_x_l and causes mitochondrial membrane depolarization in AtT20 cells. (*A*) AtT20 cells were transiently transfected with an NFκB reporter gene, and after 24 hrs treated with either vehicle or the indicated concentrations of curcumin for 4 hrs. Cells were washed and luciferase activity in cell lysates was determined. Normalized luciferase activity was calculated, and data is presented as fold change over control. Each value is the mean ±SEM of 3 separate experiments each performed in triplicates. * indicates significant difference from control, (*p<0.05*). (*B*) AtT20 cells were treated with either vehicle or indicated concentrations of curcumin for 24 hrs. Cell lysates were harvested and equal amount of protein was subjected to western blotting with an anti-Bcl-xL Ab. The filter was stripped and reprobed with anti- β tubulin Ab to confirm equal loading. Data shown is from a single experiment, and is a representative of 2 independent experiments yielding similar results. (*C*) AtT20 cells were treated with either vehicle or indicated concentrations of curcumin for 24 hrs. Cells were washed and labeled with the dual fluorescence mitochondrial specific dye, JC-1, and analyzed by flowcytometry. The dot plots show that in vehicle and 5 µM curcumin treated cells, the % of cells emitting green fluorescence is low, and is indicative of basal apoptosis. However, treatment with 50 µM curcumin, that caused a decrease in Bcl-xL levels, significantly increased the intensity of green fluorescence. The dot plot shown is from a single experiment that is representative of 3 independent experiments. When data from the light scatter plots were quantitated (*D*) as % of gated cells, our data show that curcumin in a concentration-dependent manner, lead to increased membrane depolarization. Each value is the mean ±SEM of 3 separate experiments. * indicates significant difference from control, (*p<0.05*).

**Figure 3:**
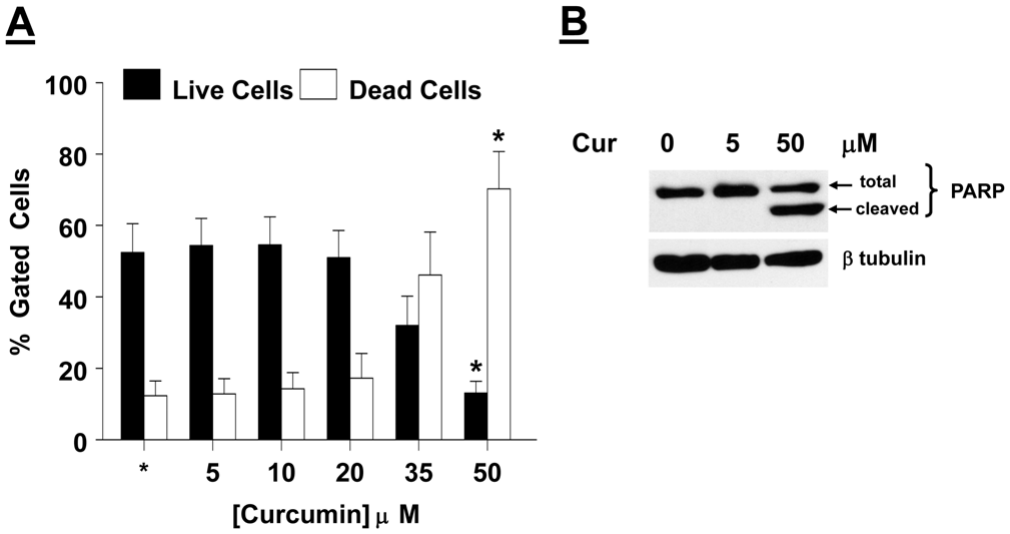
Curcumin induced apoptosis in AtT20 cells. (*A*) AtT20 cells were treated with the indicated concentration of curcumin for 24 hrs. Cell lysates were harvested and equal amount of protein was subjected to western blotting with an anti-total PARP Ab, to detect apoptosis. Membrane was stripped and reprobed with anti-β tubulin Ab to confirm equal loading. Data presented is from a single experiment, and is a representative of 3 separate experiments yielding similar results. (*B*) AtT20 cells were treated with the indicated concentration of curcumin for 24 hrs. Cell were washed and labelled with anexinV-FITC and propidium iodide and analyzed by flow cytometry. Data are presented as % of gated cells. Each value is the mean ±SEM of 3 separate determinations from a single experiment, and is a representative of 3 separate experiments yielding similar results. * indicates significant difference from control, (*p<0.05*).

We next questioned whether curcumin induced growth suppression is due to suppression of NFκB activity. To address this issue, AtT20 cells were transiently transfected with an NFκB-Luciferase reporter gene [17]. AtT20 cells were treated with vehicle or curcumin 5 and 50 µM for 4 hrs, and luciferase activity was determined. Our results (Fig. 2A) show that in AtT20 cells NFκB is constitutively active, and that curcumin inhibits this activity in a concentration-dependent manner. We next questioned whether the curcumin -induced decreased NFκB transcriptional activity would also result in decreased expression of NFκB target genes, in particular those that regulate cell survival. To address this issue, AtT20 cells were treated with either vehicle or curcumin (5 and 50 µM) for 24 hrs, and equal amount of cell lysates were examined for expression of pro-survival proteins. Our results show (Fig. 2B) that curcumin caused a decrease in the expression of pro-survival protein Bcl-xl. We next questioned whether curcumin induced decreased Bcl-xl levels would shift the balance of pro-apoptotic proteins and pro-survival proteins in favor of pro-apoptotic proteins, thereby depolarizing the mitochondrial membrane. AtT20 cells were treated with either vehicle or 5 and 50 µM curcumin for 24 hr, cells were washed and incubated with JC-1 dye (a mitochondrial-specific dual fluorescence probe) according to the manufacturer's instructions. In intact cells JC-1 dye coalesces in the mitochondria to emit a red fluorescence; however, in apoptotic cells with a depolarized mitochondrial membrane, mitochondrial contents gain entry into the cytosol, and hence JC-1 dye cannot accumulate in the mitochondria and it enters the cytosol emitting a green fluorescence. The dot plots show (Fig. 2C) that in a dose-dependent manner curcumin treatment lead to increased green fluorescence. Quantitation of the dot plots show (Fig. 3D) that in vehicle treated cells 85.82% of the cells emitted a red fluorescence while only 13.36% emitted green fluorescence. Likewise in 5 µM curcumin treated AtT20 cells, 85.34% of the cells emitted a red fluorescence while only 13.41% emitted a green fluorescence. However, in 50 µM curcumin treated cells only 31.98% cells emitted red fluorescence while 67.68% emitted green fluorescence. These results demonstrate that in a concentration-dependent manner curcumin treatment led to a significant reduction in the number of cells with intact mitochondrial membranes.

### Curcumin induced apoptosis in AtT20 cells

We next questioned whether the decreased expression of the pro-survival protein, Bcl-xl, would lead to increased apoptosis. To address this issue, AtT20 cells were treated with vehicle or curcumin (5 and 50 µM) for 24 hrs, and equal amount of cell lysates were subject to Western blotting with an anti-PARP Ab that detects both, the total as well as cleaved form of PARP. Our results show (Fig. 3A) that in a concentration-dependent manner, curcumin caused increased cleavage of PARP. Next, we confirmed whether curcumin induced apoptosis in AtT20 cells. To address this issue, AtT20 cells were treated with vehicle or curcumin (5 and 50 µM) for 24 hrs, and propidium iodide and anexin V-FITC staining was used to examine apoptosis. Our results show (Fig. 3B) that curcumin induced a decrease in the live cell population with a concomitant increase in the dead cell population.

### Curcumin decreases secretion of ACTH

**Figure 4:**
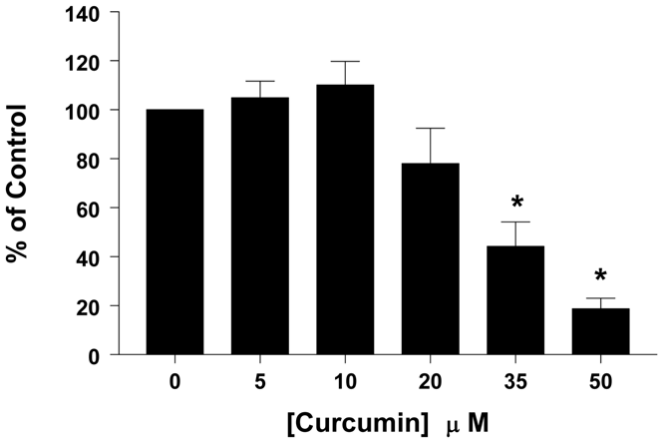
Curcumin decreases secretion of ACTH. AtT20 cells were treated with the indicated concentrations of curcumin for 24 hrs, and equal amount of CM were used to detect secreted ACTH. Data were calculated as % of vehicle control and each value is the mean ±SEM of 4 separate experiments. * indicates significant difference from control, (*p<0.001*).

We next questioned whether curcumin would have any effect on ACTH secretion in AtT20 cells. To address this issue, AtT20 cells were treated with vehicle or curcumin (5 and 50 µM) for 24 hrs, and the amount of ACTH secreted into the conditioned medium (CM) was determined. Our data show (Fig. 4) that, in a concentration-dependent manner curcumin decreased the secretion of ACTH in AtT20 cells, with significant decrease (p<0.001) being detected with concentrations as low as 35 µM. With 50 µM curcumin, maximal suppression of ACTH secretion was achieved. Note: To confirm that the observed decreases in ACTH were not due to a direct effect of curcumin on the assay, 50 µM curcumin was incubated with ACTH standard, and was found to have no effect (data not shown).

## Discussion

Approximately 15% of all intracranial tumors are of pituitary origin. Although these tumors rarely metastasize, they do cause significant pathological effects due to excess hormone secretion as well as compression of the surrounding structures. Pituitary corticotroph tumors secrete excess ACTH resulting in CD. The progression of CD is accompanied by several pathological conditions including diabetes, osteoporosis and hypertension [3]. Considering that CD was described almost 80 years ago, and to date, the standard treatment for CD is surgery followed by radiation, pharmacotherapy still remains a challenge. Recently, our laboratory demonstrated for the first time that effective growth suppression of prolactin and growth hormone producing tumor cell could be achieved by using curcumin, a natural food additive [1]. Subsequently, *Schaaf et.al.* confirmed our findings, and further demonstrated the *in vivo* ability of curcumin to suppress pituitary tumorigenesis [16]. However, the molecular mechanisms by which curcumin suppresses pituitary tumorigenesis remain unknown. In this study, we report that curcumin suppresses NFκB activity, thereby modulating the expression of pro-survival genes, and ultimately leading to mitochondrial apoptosis.

Our data show that curcumin in a concentration-dependent manner inhibited the proliferation of pituitary corticotroph tumor cells, AtT20 cells (Fig. 1A). Our observations on the growth suppressive effects of curcumin are consistent with a recent report confirming the growth inhibitory effects of curcumin on pituitary tumor cells. Although in our study, we detected growth suppression (by using the MTT assay) with 20 µM curcumin, *Schaaf et.al.* report significant growth suppression (by measuring 3[H] incorporation) with concentrations as low as 5 µM [16]. This discrepancy between the growth suppressive concentrations of curcumin could be attributed to the sensitivities of the growth assays used in the two studies. In general, the concentrations of curcumin used in both studies were similar to those used in other *in vitro* studies demonstrating the growth-suppressive effects of curcumin in different tumor types [18]–[22]. Further, our study demonstrates that the ability of curcumin to suppress corticotroph tumor cell proliferation was irreversible. When AtT20 cells were exposed to curcumin for 24 hrs, and subsequently placed in growth medium containing 10% FBS, they failed to proliferate (Fig. 1B). In addition, the ability of AtT20 cells to form colonies (Fig. 1C) was blocked in a concentration-dependent manner by curcumin, with significant inhibition detected with concentrations as low as 5 µM curcumin, and a complete blockade from concentrations of 20 µM or higher (Fig. 1C&D).

We next wanted to identify the molecular mechanism by which curcumin inhibits AtT20 cell proliferation. Previous work has shown that curcumin exerts its anti-tumor property by suppressing TNFα mediated NFκB activation. Curcumin suppresses the TNFα-induced activation of IKK that leads to the inhibition of TNF-dependent phosphorylation and degradation of IκBα and subsequent nuclear translocation of the p65 subunit of NFκB to regulate gene expression [11]. The target genes that are affected by this are several important regulators of cell cycle progression (cyclin D family), apoptosis (bcl-2), regulators of cell migration and invasion (MMP 2 and MMP-9) [11], [23], [24]. Further, constitutive activation of NFκB has been reported in cell lines as well as tumor samples and is believed to be linked to tumor progression as well as drug resistance [25]–[28]. Therefore inhibition of NFκB signaling pathway could provide an effective method to regulate pituitary tumorigenesis. Our data show (Fig. 2A) that, in a concentration-dependent manner, curcumin suppresses constitutively activated NFκB in AtT20 cells. We next questioned whether decreased NFκB transcriptional activity would result in decreased expression of target genes involved in cell survival. To this end we examined the effect of curcumin on expression of the bcl-2 family of pro-survival proteins. Our data show that in AtT20 cells we detect a robust expression of Bcl-xl, which is decreased in response to curcumin treatment (Fig. 2B). Further, we observed that curcumin treatment had no effect on the levels of bcl-2 (data not shown). Our results are consistent with a recent reports demonstrating a central role for Bcl-xl in pituitary cell survival and apoptosis [29], [30]. Although it is known that changes in intracellular levels of Bcl-xl can dramatically affect cell survival, its role in tumorigenesis has been mostly examined in tumors of the hematopoetic system [31], [32]. Our data clearly demonstrate that Bcl-xl plays a major role in regulating cell survival in pituitary corticotrophs, and manipulating its levels could provide a novel therapeutic avenue to suppress corticotroph tumor growth.

Decreased Bcl-xl levels would lead to mitochondrial membrane depolarization followed by cytochrome c release and execution of the death pathway. We observed that consistent with the decreased levels of Bcl-xl, curcumin treatment lead to mitochondrial membrane depolarization (Fig. 2C &D) and cleavage of PARP and ultimately apoptosis (Fig. 3A &B).

Excess ACTH levels and hypercortisolemia are associated with the progression of CD, and decreasing hormone levels is a therapeutic goal. We next investigated whether curcumin would have any effect on ACTH secretion in corticotrophs. Our data show that, in a concentration-dependent manner, curcumin decreased secretion of ACTH from AtT20 cells (Fig. 4). Our results showing the decreased secretion of ACTH from AtT20 cells are in agreement with similar observations reported by *Schaaf et.al.* [16], and clearly demonstrate the potential of curcumin to achieve the therapeutic goal of lowering hormone secretion.

In conclusion, we show that curcumin irreversibly inhibits proliferation, induces apoptosis and abolishes clonogenic ability of pituitary corticotroph tumor cells. The induction of apoptosis is accompanied by decreased NFκB transcriptional activity and expression of the pro-survival protein Bcl-xl. We also demonstrate that curcumin suppresses ACTH secretion from pituitary corticotroph tumor cells. Based on this we propose developing curcumin as a novel therapeutic agent in the management of CD.

## Materials and Methods

### Chemicals and reagents

Curcumin was purchased from LTK laboratories (St. Paul, MN). Anti -total PARP and -bcl_x_l antibodies were purchased from Cell Signaling Technologies, (Danvers, MA). Horseradish peroxidase (HRP)-conjugated secondary antibodies were purchased from Upstate Biotechnology (Lake Placid, NY).

### Cell culture

AtT20 cells were a kind gift by Dr. Pamela Mellon's laboratory (University of California San Diego, La Jolla, CA). Cells were maintained in complete medium [DMEM (Mediatech, Manassas, VA) containing 10% FBS (Gibco/Invitrogen, Grand Island, NY) and 5 U/ml Penicillin/5 µg/ml Streptomycin]. Medium was changed every 2–3 days, and sub-culturing was as required.

### Assessment of cell proliferation

AtT20 cells in log phase were seeded (30,000 cells/well) on 96 well plates in complete medium (containing 10% FBS). After 24 hrs, cells were treated with curcumin in medium containing 0.5% FBS. Proliferation was assessed using the colorimetric MTT assay as described previously [33].

### Colony formation assays

Colony formation assays were performed as described [34]. Briefly, AtT20 cells growing in log phase were seeded (1000–3000 cells/well in a 6 well plate) in growth medium (containing 10% FBS). After allowing the cells to adhere for 24 hrs, medium was replaced with fresh growth medium containing the indicated concentrations of curcumin. Cells were incubated at 37°C for 14–21 days, with medium changes (including curcumin) every fourth or fifth day. Crystal violet staining was used to visualize colonies.

### Mitochondrial membrane depolarization assays

AtT20 cells were treated with the indicated amount of curcumin for 24 hrs. According to the manufacturer's protocol (Cell Technologies Inc. Mountain View, CA) cells were washed, stained with JC-1 dye, and then analyzed by flow cytometry.

### Annexin V staining assays

Was performed as described before [1]. Briefly, AtT20 cells were treated with the indicated amount of curcumin for 24 hrs. This was followed by staining with annexin V - FITC and propidium iodide, and analysis by flow cytometry.

### Western blotting

After the indicated treatment, cell lysate preparation, protein content determination and western blotting was performed as described previously [1].

### NFκB/Luciferase reporter gene assays

AtT20 cells were plated in 24 well plates, and 24 hrs later cells were transfected with 0.8 µg of NFκB/Luciferase plasmid [17] (gift from Dr. S.C. Gautam, Detroit, MI); using Lipofectamine 2000 (Invitrogen) according to the manufacturers instruction. After 18–24 hrs, medium was replaced with plating medium containing the indicated concentration of curcumin. After 4 hrs cells were lysed and luciferase activity was determined using a commercial Luciferase assay kit (Promega, Madison, WI). Fold change in NFκB-luc activity was calculated after normalization of light units to µg of cell protein.

### Measurement of ACTH

AtT20 cells were treated with the indicated concentrations of curcumin for 24 hrs, and conditioned medium (CM) was harvested, and ACTH concentration was determined by using a commercial ACTH-RIA kit (MP Biomedicals, Solon, OH) according to the manufacturer's instructions.

### Data analysis

Statistical significance was determined using Student's *t* test or one-way analysis of variance followed by Duncan's multiple range test as required.
