# Correction: Growth Suppression of Mouse Pituitary Corticotroph Tumor AtT20 Cells by Curcumin: A Model for Treating Cushing's Disease

**DOI:** 10.1371/annotation/38a101d6-a1f2-4a74-ab63-bc5c61e5f62b

**Published:** 2010-04-30

**Authors:** Madhavi Latha Yadav Bangaru, Jeffrey Woodliff, Hershel Raff, Sanjay Kansra

Affiliations 1 & 2 are incorrect. Affiliation number 1 should read: Department of Endocrinology, Metabolism & Clinical Nutrition, Medical College of Wisconsin, Milwaukee, Wisconsin, United States of America. Affiliation number 2 should read: Department of Pediatrics, Medical College of Wisconsin, Milwaukee, Wisconsin, United States of America.

